# An Unusual Canopy Ascent by a Grass Snake (*Natrix natrix*) to a Crow Nest

**DOI:** 10.1002/ece3.74717

**Published:** 2026-11-13

**Authors:** Valentine Comin, Guillam E. McIvor, Jérémy Lemaire, Rosanna Mangione, Barbara C. Klump

**Affiliations:** ^1^ Department of Behavioral and Cognitive Biology University of Vienna Vienna Austria; ^2^ Haidlhof Research Station University of Vienna and University of Veterinary Medicine Vienna Bad Vöslau Austria

**Keywords:** arboreal, climbing ability, predation, reptile

## Abstract

Grass snakes (*Natrix natrix*) are a semi‐aquatic colubrid species that are seldom observed beyond ground level. While previous notes have reported the species occasionally climbing shrubs and small trees to either bask or prey on arboreal species, we present evidence of a live individual that was identified at a height of 16.7 m in an empty crow nest in an oak tree during a drone survey. This presents a substantial increase on the previous record for the species of 6 m and we discuss potential sensory, morphological, and behavioral explanations for why the snake was so high in the tree. Our observation highlights the individual flexibility that may be overlooked by standard surveys, guiding future behavioral and ecological studies.

## Graphical abstract

We present evidence of a live grass snake (*Natrix natrix*) at a height of 16.7 m in an empty crow nest, over 10 m higher than previous reports. We discuss potential sensory, morphological, and behavioral explanations for why the snake was so high in the tree. Our observation was made possible by a drone, highlighting the individual flexibility that may be overlooked by standard surveys, guiding future behavioral and ecological studies.

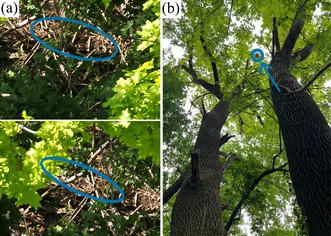

1

Grass snakes, *Natrix natrix* (Linnaeus 1758), have been reported climbing shrubs and trees to sun bask or prey on arboreal species. To our knowledge, the highest a grass snake has been reported climbing was 6 m by Vaughan (2008), who observed a female grass snake climbing through tree branches after breaking away from a mating ball. The males later joined the female in the tree and continued mating.

On the 27th of April 2026, the authors were monitoring active crow nests ( *Corvus corone* spp.) using a drone (model DJI M30T) in the region of Traiskirchen, Austria. This area flanks the Schwechat river and has a high density of crow nests. The nest in question was in an oak tree, *Quercus* spp. (48.02196° N, 16.31790° E) at a height of 16.7 m (as measured with a Bosch DLE50 laser range finder) at a distance of around 90 m from the river. The nest was found empty except for a grass snake, *Natrix natrix* (Figure 1a), which was alive and moving around the nest (embedded Video 1). The crow nest was situated on a branch at about 16.7 m high (Figure 1b, estimated with a Bosch DLE 50 laser range finder). The nest was originally found by GEM on the 18th of March and monitored by GEM and VC every 3–9 days thereafter (5 days on average). A female crow was observed sitting on the nest on the 30th of March 2026 suggesting that a clutch had been initiated and incubation had started. Based on the incubation period of 18 days in crows (Loman 1980) and monitoring of the local population in previous years, we expected chicks to hatch around the 17th of April, although we did not notice clear signs of hatching (e.g., feeding by the parents) around that time. On the 27th of April, GEM and VC arrived in the area around 12 p.m. to monitor nearby nests nearby and the target nest was observed with the drone around 4:30 p.m.

**FIGURE 1:**
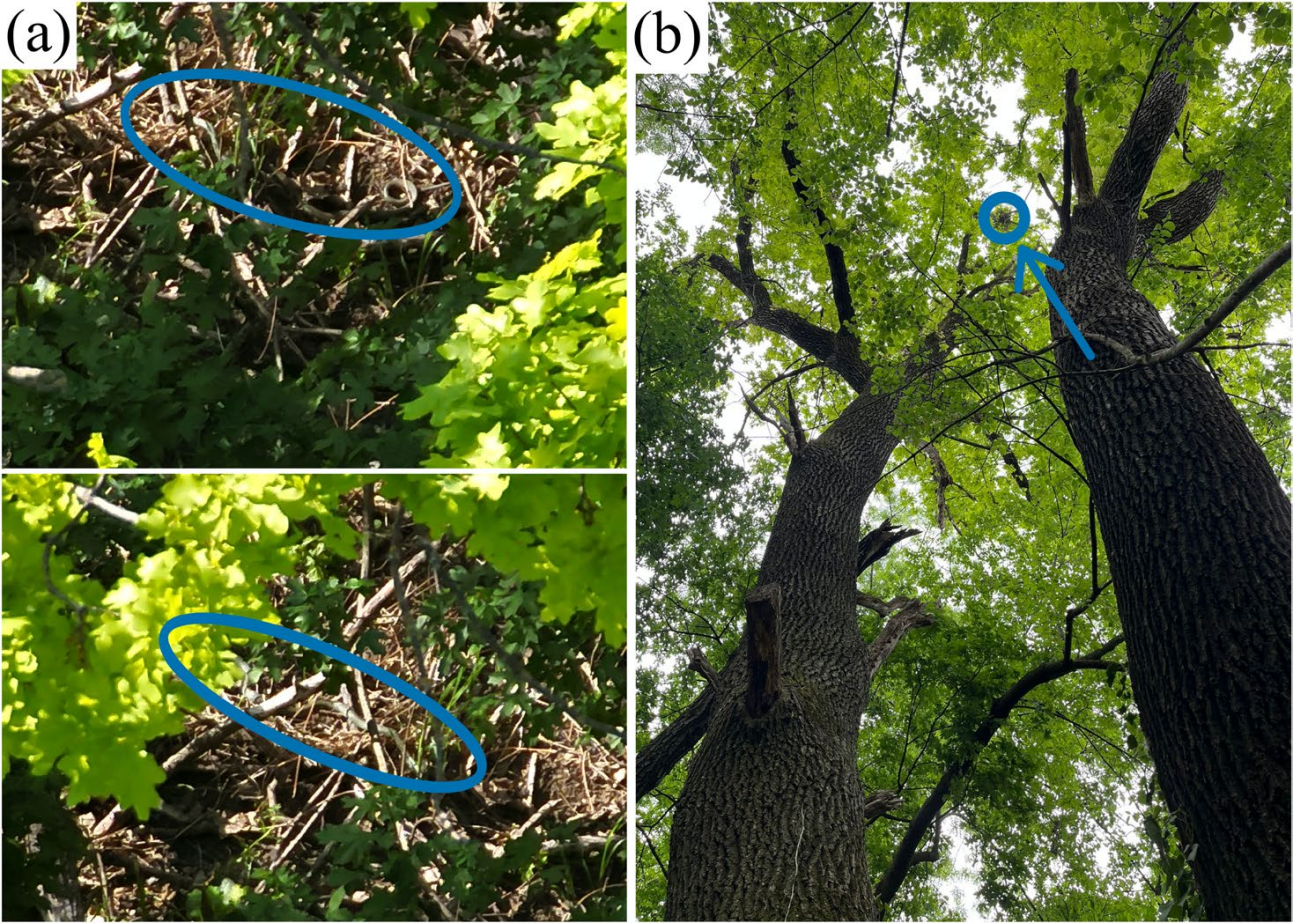
(a) Drone photos of the grass snake in the nest. The snake is circled. The photos were taken using the drone's zoom camera (focal length: 21–75 mm; equivalent: 113–405 mm; aperture: F/2.8–f/4.2). (b) Photo of the oak tree, showing the few branches available for the climb and the slope of the trunk from 5 m upwards. The nest is circled.

**VIDEO 1:** Drone footage showing a grass snake, *Natrix natrix* , moving in an empty crow nest. Video content can be viewed at https://onlinelibrary.wiley.com/doi/10.1002/ece3.74717.

Our observation raises two questions: how did the snake climb to the nest, and why did it do so? Because no grass snake has been reported this high, and we found the snake in an empty nest, we can only speculate about the answers.

We believe that the snake climbed the tree on its own rather than being brought to the nest by a bird of prey, since it was alive and appeared healthy, which suggests that grass snakes may be more agile climbers than is generally assumed. Nevertheless, the height to which the snake climbed remains highly unusual based on what has previously been described for the species. The bottom part of the trunk on the oak tree is straight with a circumference of 217 cm at the base. Only about 10 dead branches are present before the first major split at 7.7 m. A fallen tree leaning against the trunk at approximately 4 m high could have served as a ramp to assist initial access (Figure 2a), and the rough texture of the bark likely helped the ascent (Figure 2b). From about 5 m upward, the trunk grows at an angle to the nest at 16.7 m. Previous reports described grass snakes climbing trees with rough bark and on a sloped tree trunk with multiple branches that facilitate climbing (Bringsøe and Aastrup 2017). Zimmermann (1909, as cited in Bringsøe and Aastrup 2017) also described the grass snake's ability to climb shrubs and small trees by sliding upwards rather than by coiling around the branches.

**FIGURE 2:**
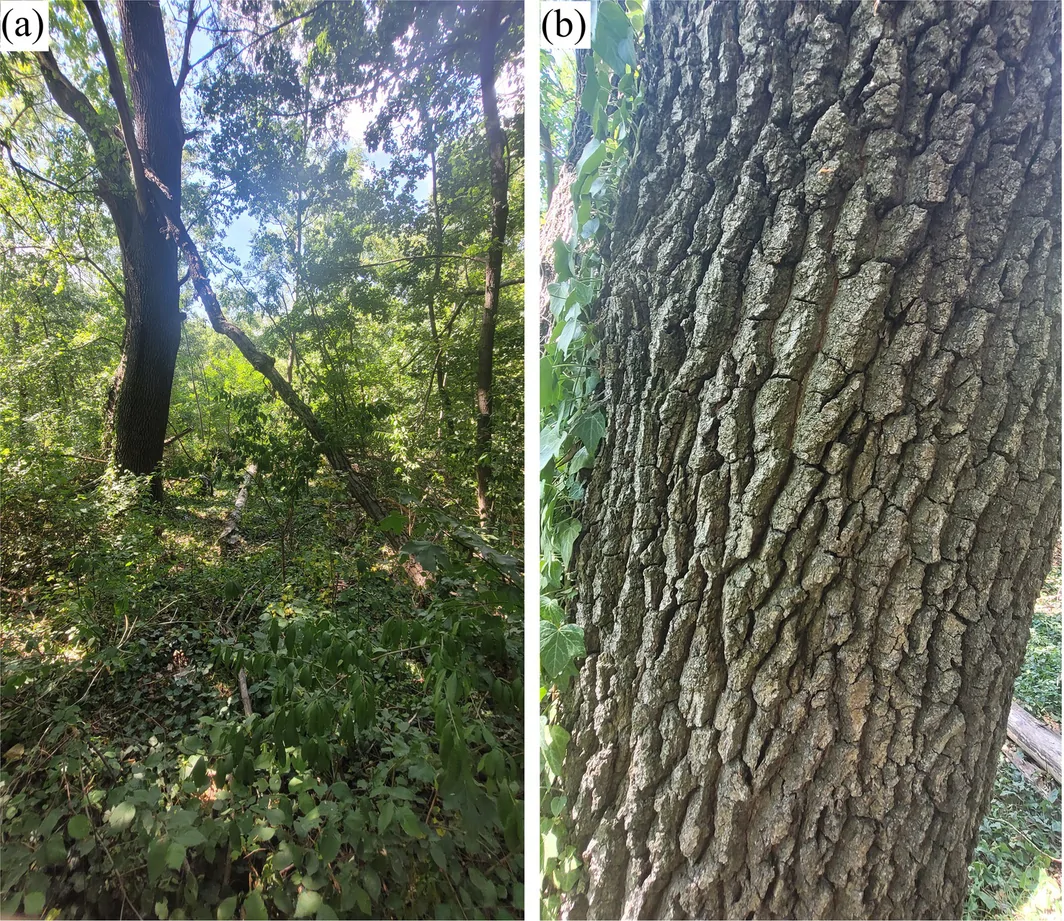
(a) View of the fallen tree, which may have served as a ramp. (b) Close‐up of the textured bark of the oak tree.

When considering why the grass snake made such an ascent, the species is known to climb bushes to bask or to prey on tree frogs and nestlings. They have been observed climbing willows (*Salix* spp.) up to about 2 m to prey on European tree frogs, *Hyla arborea* (Kaczmarski 2020), and taking nestlings from a shrub nest above water (Rollinat 1934). However, in our case, the nest was much higher, making it unlikely that the snake climbed with the specific intent of preying on eggs or hatchlings. Although grass snakes are active hunters, their ability to detect prey from a distance from vibrations (Young 2003), visually (Land and Nilsson 2012) or through olfaction (Schwenk 1995) is limited. Furthermore, we observed none of the disturbances typically associated with a predation event (e.g., crow alarm calling, mobbing, or agitated flight). We find it unlikely that predation occurred before we arrived on site, as snakes usually leave the predation site shortly after feeding (Greene 1997; DeGregorio et al. 2016). We also find it unlikely that the snake climbed the tree to bask since a large log pile, making a suitable basking site at a much lower height, was available about 40 m away from the tree (Figure 3).

**FIGURE 3:**
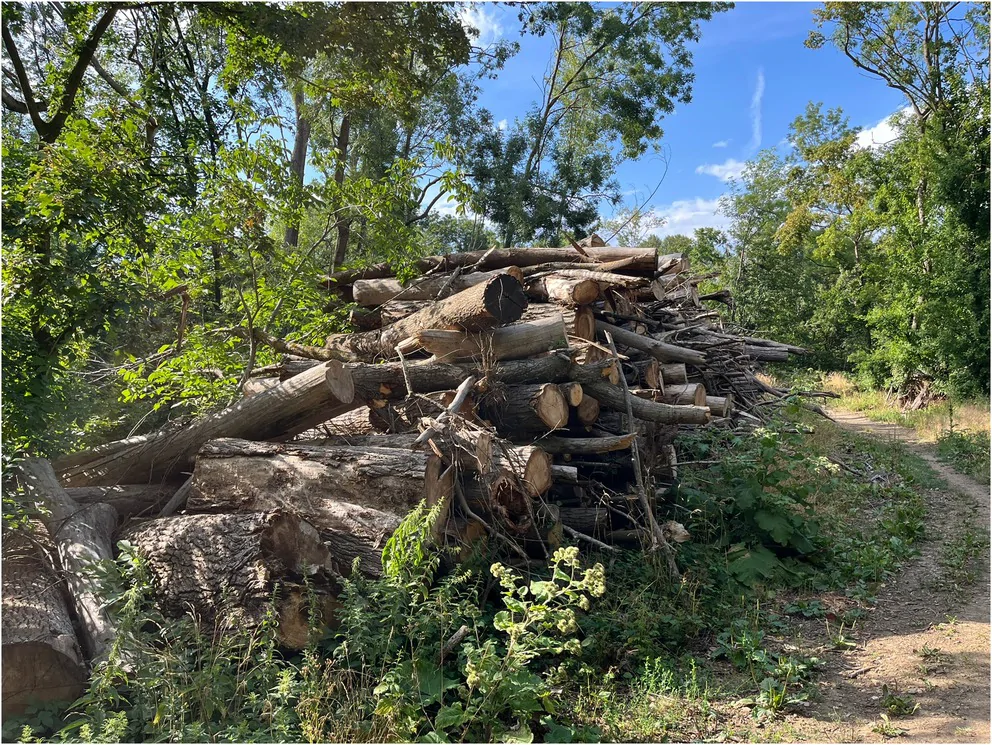
Photo of the log pile, providing a suitable basking site about 40 m from the tree.

Our observation of a live grass snake in a crow nest about 16.7 m above the ground reflects the snake's behavioral flexibility or capacity for innovation. Studies of nest predation show that a small number of identifiable snakes can account for a large share of nest predation (DeGregorio et al. 2016), pointing to individual specialization in foraging practices. While caution should be applied to not over‐generalize from a single case, with the advancements in drone technologies over the last decade (Anderson et al. 2025), we expect to see an increase of such observations in the future, helping us document and understand aspects of a species' behavior and physical capacities that would otherwise go unnoticed.

## Author Contributions

**Valentine Comin:** writing – original draft (lead), writing – review and editing (equal). **Guillam E. McIvor:** supervision (equal), writing – review and editing (equal). **Jérémy Lemaire:** writing – review and editing (equal). **Rosanna Mangione:** writing – review and editing (equal). **Barbara C. Klump:** funding acquisition (lead), project administration (lead), supervision (equal), writing – review and editing (equal).

## Funding

This work was supported by the Austrian Science Fund (10.55776/ESP408) and the Vienna Science and Technology Fund (10.47379/VRG21011).

## Ethics Statement

Crow nest monitoring was conducted under licenses issued by the Animal Ethics and Experimentation Board at the Faculty of Life Sciences, University of Vienna, Austria (application no. 2023‐033) and the Bezirkshauptmannschaft Baden, Austria (BNL2‐J‐082/116).

## Conflicts of Interest

The authors declare no conflicts of interest.

## Data Availability

All original data are presented in the manuscript.
